# Sudden Cardiac Death Caused by a Fatal Association of Hypertrophic Cardiomyopathy (*MYH7*, p.Arg719Trp), Heterozygous Familial Hypercholesterolemia (*LDLR*, p.Gly343Lys) and SARS-CoV-2 B.1.1.7 Infection

**DOI:** 10.3390/diagnostics11071229

**Published:** 2021-07-07

**Authors:** Nicola Marziliano, Alessandro Medoro, Donatella Mignogna, Giovanni Saccon, Stefano Folzani, Claudio Reverberi, Claudio Russo, Mariano Intrieri

**Affiliations:** 1 Department of Medicine and Health Sciences “V. Tiberio”, University of Molise, 86100 Campobasso, Italy; alessandro.medoro@unimol.it (A.M.); donatella.mignogna@studenti.unimol.it (D.M.); g.saccon@studenti.unimol.it (G.S.); claudio.russo@unimol.it (C.R.); intrieri@unimol.it (M.I.); 2 Clinical Pathology Laboratory, ASST Rhodense, Rho, 20017 Milan, Italy; 3 Poliambulatorio Città di Collecchio, Collecchio, 43044 Parma, Italy; folzanimed@gmail.com (S.F.); claudioerre110153@gmail.com (C.R.)

**Keywords:** hypertrophic cardiomyopathy, familial hypercholesterolemia, *MYH7*, *LDLR*, SARS-CoV-2, lineage B.1.1.7, fatal arrhythmia

## Abstract

Hypertrophic cardiomyopathy (HCM) and heterozygous familial hypercholesterolemia (HeFH), two of the most common genetic cardiovascular disorders, can lead to sudden cardiac death. These conditions could be complicated by concomitant severe acute respiratory syndrome coronavirus 2 (SARS-CoV-2) infection as in the case herein described. A young amateur soccer player died in late October 2020 after a fatal arrhythmia and the autopsy revealed the presence of HCM with diffuse non-obstructive coronary disease. The molecular autopsy revealed a compound condition with a first mutation in the *MYH7* gene (p.Arg719Trp) and a second mutation in the *LDLR* gene (p.Gly343Cys): both have already been described as associated with HCM and HeFH, respectively. In addition, molecular analyses showed the presence of SARS-CoV-2 lineage B.1.1.7 (UK variant with high titer in the myocardium. Co-segregation analysis within the family (*n* = 19) showed that heterozygous *LDLR* mutation was maternally inherited, while the heterozygous *MYH7* genetic lesion was de novo. All family member carriers of the *LDLR* mutation (*n* = 13) had systematic higher LDL plasma concentrations and positive records of cardiac and vascular ischemic events at young age. Considering that HCM mutations are in themselves involved in the predisposition to malignant arrhythmogenicity and HeFH could cause higher risk of cardiac complications in SARS-CoV-2 infection, this case could represent an example of a potential SARS-CoV-2 infection role in triggering or unmasking inherited cardiovascular disease, whose combination might represent the cause of fatal arrhythmia at such a young age. Additionally, it can provide clues in dating the presence of the SARS-CoV-2 lineage B.1.1.7 in Northern Italy in the early phases of the second pandemic wave.

## 1. Introduction

Hypertrophic cardiomyopathy (HCM; MIM #192600) and heterozygous familial hypercholesterolemia (HeFH; MIM #144010) are the most common genetic cardiovascular disorders with a respective carrier frequency of ~1:500 and ~1:200–300 [1,2]. HCM is defined by increased left ventricular wall thickness or mass that is not solely explained by increased loading conditions such as in sports activities. HCM causes are primarily due to mutations in cardiac sarcomeric protein genes and is inherited in an autosomal dominant manner: mutations in the genes encoding for beta-myosin heavy chain (*MYH7*) and myosin-binding protein C (*MYBPC3*) account for the majority of cases [3]. On the other hand, HeFH is characterized by raised serum LDL cholesterol level resulting in excess deposition of cholesterol in tissues and arterial walls, leading to accelerated atherosclerosis and increased risk of premature coronary heart disease. HeFH’s most common causes are pathogenic variants of the LDL receptor gene (*LDLR*), which are responsible for 85–90% of genetically confirmed HeFH [4]. Although the phenotypic spectrum can be extremely different, both HCM and HeFH can lead to sudden cardiac death caused by malignant ventricular arrhythmias and severe heart failure or coronary artery disease and ischemic heart disease, respectively.

Considering the severe acute respiratory syndrome coronavirus 2 (SARS-CoV-2) pandemic, the above-mentioned cardiovascular conditions could be complicated by concomitant SARS-CoV-2 infection. Indeed, it has been ascertained that SARS-CoV-2 infection could be the cause of the increasingly recognized cardiovascular complicacies. The relationship among cardiac pathological changes, cardiac infection by SARS-CoV-2, and clinical features has not yet been fully elucidated and is likely multifactorial. Multiple manifestations of cardiac pathology have been described in patients with fatal SARS-CoV-2 infections, such as microvascular thrombi, ischemic injury, right ventricular strain injury, pericarditis, myocardial inflammatory cellular infiltrates, and full myocarditis with myocyte destruction [5,6]. Furthermore, patients with established cardiovascular disease have an increased risk for severity and mortality with SARS-CoV-2 infection, mainly due to ACE2 receptors abundance in the cardiovascular system, which represents the gateway for the virus entry in the heart [7]. Although still debated, acute SARS-CoV-2 induced myocarditis represents one of the most common cardiovascular SARS-CoV-2 complicacy along with other pathogens with cardiac tropism [8,9]. Therefore, acute myocarditis presents across a variable range of clinical severity and is a significant diagnostic challenge in the SARS-CoV-2 infection era, considering that diffuse myocardial injury was also detected in the early convalescent stage of SARS-CoV-2 infected and recovered patients who had no active cardiac symptoms [10]. Indeed, its pathophysiology is thought to be a combination of direct myocardial cell injury via ACE2 receptors, cardiac damage due to the host immune response as an effect of cytokine storm (TNF-α and interleukin-6 driven), affecting multiple organ systems, and myocardial oxygen supply/demand mismatch leading to cardiac manifestations, such as acute heart failure and arrhythmia [11,12].

In this report, we describe a successful interdisciplinary workout between cardiologists, clinical pathologists and geneticists in the identification of two pathogenic mutations in *MYH7* (p.Arg719Trp) and *LDLR* (p.Gly343Cys) genes, associated with HCM and HeFH. respectively, in a young man who suddenly died after a fatal arrhythmia who, during autopsy, tested positive for SARS-CoV-2 virus with high titer in the myocardium (threshold cycle values below 25) and belonging to the lineage B.1.1.7 (UK variant). This latter finding provides an initial snapshot of SARS-CoV-2 genomic diversity during the early phases of the second pandemic wave in Northern Italy dating the presence of the SARS-CoV-2 lineage B.1.1.7 in the late October 2020.

## 2. Case Report

The proband was a young man (32-year-old, body mass index: 23.24) who suddenly died during physical activity. He was an amateur soccer player and during his last match in late October 2020 he fainted. All the witnesses said that he appeared in good health and that he suddenly collapsed without any previous symptoms.

An immediate on-site 12-leads electrocardiogram (ECG) documented a fatal sustained ventricular arrhythmia (ventricular fibrillation) with signs of inverted T-waves. The patient unsuccessfully underwent cardiac resuscitation and eventually died upon arrival at the hospital. The proband had never suffered from cardiovascular disease before this fatal event nor were signs of ongoing flu/flu-like symptoms disclosed by his family members. The public prosecutor requested an autopsy to verify the cause of the death. The autopsy was performed 2 d after death. At external examination of the body no relevant signs were evidenced. The whole heart was washed under running water for roughly 1 h and the fixed in buffered 10% formalin overnight. After fixing, longitudinal and transversal cuts were done in order to respectively obtain a 4- and 2-chambers view of the inner heart.

**Figure 1:**
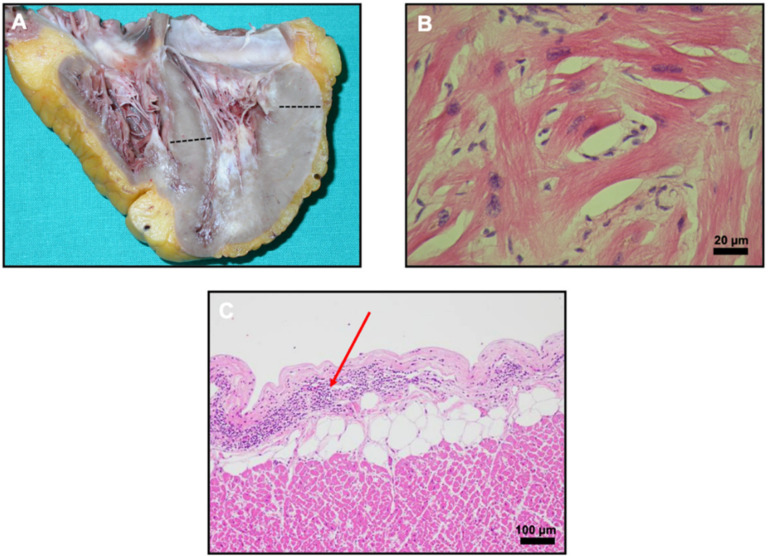
(**A**) Two chambers view of the proband’s heart. The increased thickness of the left lateral ventricle wall (2.0 cm) and the inter ventricular septum (1.8 cm) is evidenced by black dotted lines. (**B**) Hematoxylin-eosin staining of the myocardium shows cardiomyocytes disarray which is often seen in hypertrophic cardiomyopathy (HCM). (**C**) Epicardium exhibits a focus with lymphocytic infiltrate (dark purple dots; red arrow) indicative of lymphocytic myocarditis.

Macroscopic heart examination showed increased heart weight of 550 g (normal ranges are up to 300 g), longitudinal diameter: 13.5 cm, transverse diameter: 9.8 cm (Figure 1A) and increased cardiac wall thickness of both interventricular septum (1.8 cm) and left posterior/lateral wall (~2.0 cm). Atria, valves and right ventricle (wall thickness: 0.9 cm) did not show any macroscopic relevant findings. Non-obstructive early diffuse coronary artery disease was also observed. Histopathological examination of the myocardium, after hematoxylin-eosin stain, showed patchy myocardial disarray, cardiomyocytes with moderately enlarged hyperchromatic nuclei and individual cardiomyocytes with vacuolar degenerative changes (Figure 1B). Inflammatory lymphocytic infiltrate indicative of myocarditis was present (Figure 1C). Microscopic evaluation of the lung tissue evidenced the presence of pulmonary thromboembolism; microthrombi were identified on light microscopic examination, along with strands of precipitated fibrin and entrapped neutrophils within alveolar capillaries as well as larger deposits of fibrin in alveolar spaces. All these autopsy findings prompted the search of cardiotropic viral particles within the myocardium, in particular SARS-CoV-2, considering the pandemic situation at the time of the autopsy.

**Figure 2:**
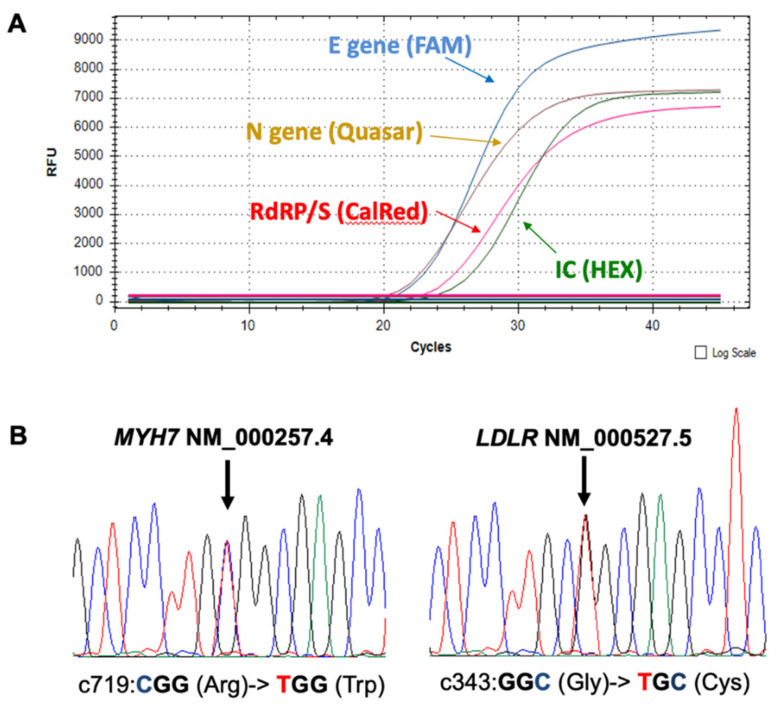
(**A**) Real time PCR (RT-PCR) shows the positivity of the proband for all the three SARS-CoV-2 genes (threshold cycle values below 25). RFU = relative fluorescent units. (**B**) Electropherograms show the identification of a heterozygous genetic variant in the *MYH7* (p.Arg719Trp) gene and a missense heterozygous mutation in the *LDLR* (p.Gly343Cys) gene in the proband.

Myocardium specimen of the interventricular septum (10 mm^3^) dissected under stereomicroscope to avoid any vessel contamination was processed for whole RNA/DNA extraction. It tested positive for a high concentration of SARS-CoV-2 particles (Figure 2A) by using real-time PCR (RT-PCR) performed with Allplex 2019-nCoV (Seegene, Inc., Seoul, South Korea) on a CFX platform (Bio-Rad, Hercules, CA, USA; Ct = 13 for Rdrp/Spike, Envelope and Nucleocapsid SARS-CoV-2 genes, respectively), suggesting either the presence of a viremic phase and a direct role in cardiotoxicity or, alternatively, infected macrophage migration from the lung carrying virus particles. Subsequently, we further proceeded with a first level of genotyping by means of the RT-PCR Seegene Allplex SARS-CoV-2 Variants I Assay (Seegene, Inc., Seoul, South Korea). The co-presence of both HV 69–70 deletion and N501Y single nucleotide polymorphism on the SARS-CoV-2 S gene prompted further next-generation sequencing (NGS) genotyping. Libraries were prepared with the Ion AmpliSeq Library Kit Plus according to the manufacturer’s instruction using Ion AmpliSeq SARS-CoV-2 RNA custom primers panel (ID: 05280253, ThermoFisher Scientific, Waltham, MA, USA). The RNA library preparation included reverse transcription using SuperScript VILO cDNA Synthesis Kit (ThermoFisher Scientific, Waltham, MA, USA) with 16–21 subsequent cycles of PCR amplification on Ion Chef (ThermoFisher, Scientific, Waltham, MA, USA). NGS reactions were run on Ion Torrent GeneStudio S5 sequencer (ThermoFisher Scientific, Waltham, MA, USA).

Sequence alignments to the SARS-CoV-2 isolate Wuhan-Hu-1 complete genome (NCBI nucleotide collection, accession number: NC_045512) [13] was performed within the Torrent Server of Ion Torrent S5 sequencer using default settings. Sequence comparison on GISAID (www.gisaid.org, accessed on 3 March 2021) platform revealed the presence of the SARS-CoV-2 lineage B.1.17 (UK variant).

Molecular autopsy was also performed by means of NGS applying a custom cardio-panel of 60 genes covering cardiomyopathies, channelopathies, collagen and metabolic disorders designed by means for the AmpliSeq chemistry (ThermoFisher, Scientific, Waltham, MA, USA) (Table 1). After sequence analysis on the Ion Torrent suite (ThermoFisher, Waltham, MA, USA) the bam files were analyzed on the BaseSpace suite (Illumina, San Diego, CA, USA). We identified a heterozygous genetic variant in the *MYH7* (p.Arg719Trp) gene and a missense heterozygous mutation in the *LDLR* (p.Gly343Cys) gene (Figure 2B), both classified as pathogenic in accordance with the American College of Medical Genetics and Genomics (ACMG) criteria [14]. Indeed, these two mutations are already described as responsible for HCM and HeFH, respectively [15,16].

**Figure 3:**
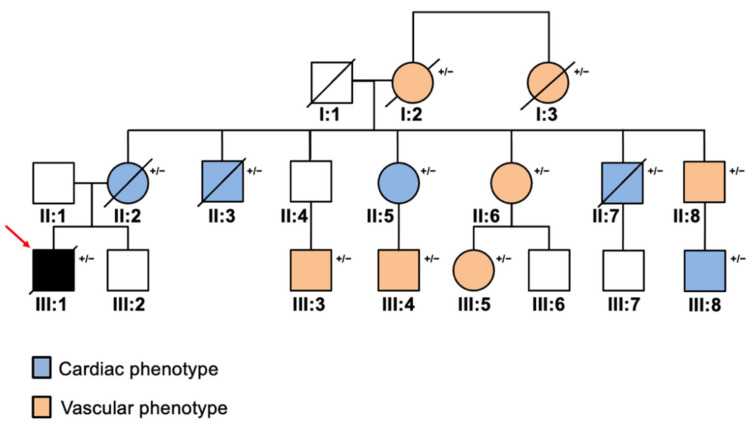
Pedigree of the family with heterozygous familial hypercholesterolemia (HeFH) (*n* = 19). Squares indicate males and circles represent females, while slashed symbols mean deceased members. The proband (III:1) is marked by a red arrow. +/− indicates the presence of *LDLR* (p.Gly343Cys) mutation.

Co-segregation analysis via Sanger sequencing (cycle sequencing kit v 3.1 on an ABI Prism 3500, Applied Biosystems, Waltham, MA, USA) within the family (*n* = 19) showed that *LDLR* mutation was maternally inherited, with 13 family members being carriers of the *LDLR* mutation, while *MYH7* genetic lesion was *de novo* (Figure 3).

All family member carriers of the *LDLR* mutation (p.Gly343Cys, rs730882096; HGMD:CM920442) had systematic LDL plasma concentrations above the 95th percentile (>130 mg/dL) and positive records of cardiac (coronary artery disease, *n* = 8) and vascular ischemic events (external carotid occlusion, *n* = 5) at young age (<45 years for women and <50 years for men) as reported in detail in Table 2. Six patients of this family, including the proband, died due to cardio-vascular complications.

## 3. Discussion and Conclusions

The case herein described represents an example of a potential SARS-CoV-2 infection role in triggering or clinically unmasking inherited cardiovascular disease, whose combination might explain the cause of the patient’s exitus. After all, there is an increasing awareness of the involvement of host genetic factors in infectious diseases, in particular in the SARS-CoV-2 era; SARS-CoV-2 infection with its variants could represent a concrete risk of worsening patient clinical status, especially in the concomitance with other congenital disease, underlying as these patients could be considered a potentially vulnerable patient cohort.

In detail, in this case, autopsy findings revealed a fatal combination of cardiac manifestations caused by HCM (left ventricle hypertrophy and cardiomyocytes disarray), HeFH (early diffuse coronary artery disease) and SARS-CoV-2 UK variant infection (lymphocytic myocarditis). Although several cases of acute myocarditis have been reported [17,18,19,20,21,22,23], none of the patients in these reports had such a distinctive genetic background or suddenly died without any kinds of symptoms attributable to the cardiac alteration or the SARS-CoV-2 infection.

However, it is not possible to assess which was the exact cause of cardiac arrhythmogenic activity described in this report. It is important to underline that SARS-CoV-2 could have a peculiar cardiac tropism, as revealed by cases in which the virus has been detected in the heart and not by nasopharyngeal swabs and distal bronchoalveolar lavage [18,24]. Indeed, several SARS-CoV-2-induced myocarditis reports have been increasingly recognized showing extended myocardial lymphocytes infiltration [18,25,26]. Myocardial injury directly linked to SARS-CoV-2 myocardial localization is more frequently observed in severe cases and it is associated with high-sensitivity troponin and higher inflammatory burden that could be responsible for vascular inflammation, myocarditis and cardiac arrhythmias [27,28]. Experimental findings showed that human induced pluripotent stem cells-derived cardiomyocytes treated with interleukins and infected with SARS-CoV-2 have increased release of troponin, disorganization of myofibrils and changes in beating, these latter two traits also present in HCM pathology [29]. Furthermore, a pivotal role has recently been proposed of severe inflammatory response during SARS-CoV-2 infection with significant proinflammatory cytokines release (predominantly interleukin-6) in the predisposition to ejection fraction reduction and other symptoms of cardiac dysfunction including arrythmias, hypotension and tachycardia [30,31]. Nevertheless, few analyses specifically compared viremic patients dying of SARS-CoV-2 infection; in particular, it has been shown in a cohort of critically ill patients with SARS-CoV-2 infection that serum interleukin-6 levels positively correlate with the severity of the disease [32]. In this scenario, it is also necessary to consider the ever-increasing number of other cardiac complications caused by the “cytokine storm” (e.g., takotsubo syndrome) that may occur during SARS-CoV-2 infection and could trigger the underlying patient’s clinical condition [33].

All these observations support our hypothesis that the electrical remodeling associated with a genetic substrate and the concomitant presence of SARS-CoV-2-induced myocarditis can trigger and potentiate the fatal arrhythmia, considering that sudden cardiac death in HCM is mainly caused by malignant ventricular arrythmias in itself [34]. Additionally, we cannot exclude a peculiar role of SARS-CoV-2 lineage B.1.1.7.

In this light, the *LDLR* p.Gly343Cys mutation should not be a simple by-stander, but a stronger phenotype modulator as already seen in Tunisian and French populations [16]. Furthermore, HeFH patients with SARS-CoV-2 infection may be at higher risk of cardiac complications compared with non-HeFH patients, particularly if the underlying genetic disease has remained undetected [35].

Considering the minimal understanding of this infection role in cardiac pathogenesis, this case highlights the importance of autopsies and post-mortem genetic analyses to unravel hidden pathogenetic mechanisms related to SARS-CoV-2 infection. Several other reports have revealed post-mortem findings of fatal associations of channelopathies and cardiomyopathies [36], but-to our knowledge-this is the first report in which a cardiomyopathy with a metabolic disorder in the presence of SARS-CoV-2 lineage B.1.1.7 infection led to a fatal outcome. Thus overall, our work: (a) strengthens the knowledge for monitoring patients with congenital cardiovascular pathologies, such as HCM and HeFH, revealing as SARS-CoV-2 infection could be detrimental; and (b) provides an initial snapshot-to our knowledge-of early SARS-CoV-2 genetic variants circulation (lineage B.1.1.7) in the second pandemic wave (late October 2020) in Northern Italy.

Despite the outcome, the continued investigation throughout the use of cardiological and genetic evaluations with molecular autopsy leads to the identification of the underlying arrhythmic mechanism. This finding is of paramount importance for the first-or second-degree relatives in which the identification of the pathogenic substrate, which renders them vulnerable to an increased risk for life-threatening cardiac events, including sudden death, might prompt for clinical and tailored treatments.

## Figures and Tables

**Table 1:** Genes included in the custom cardio-panel covering cardiomyopathies, channelopathies, collagen and metabolic disorders.

Genes Included in the Custom Cardio-Panel
*ACTC1, ACVRL1, APOB, BAG3, BMPR2, BRAF, CACNA1C, CASQ2, DES, DMD, DSC2, DSG2, DSP, ELN, EMD, ENG, FBN1, FLNC, GATA4, GLA, JAG1, JUP, KCNE1, KCNE2, KCNH2, KCNJ2, KCNJ8, KCNQ1, KRAS, LAMP2, LDLR, LDLRAP1, LMNA, MYBPC3, MYH7, MYL2, MYL3, NF1, NKX2-5, PKP2, PLN, PRKAG2, PCSK9, PTPN11, RAF1, RBM20, RYR2, SCN1B, SCN5A, SOS1, SOS2, TAZ, TGFBR2, TMEM43, TNNC1, TNNI3, TNNT2, TPM1, TTN, TTR*

**Table 2:** Clinical and genetic data of proband family members with cardiac and vascular phenotype. Proband is highlighted in bold.

ID	Age	Sex	Genotype	sLDL Levels (mg/dL) *	Phenotype	
					Cardiac	Vascular
I:2	50 ^†^	F	*LDLR* +/−	182	CAD	-
I:3	55 ^†^	F	N.A.	210	CAD	-
II:2	60 ^†^	F	*LDLR* +/−	197	-	ECO
II:3	58 ^†^	M	*LDLR* +/−	221	-	ECO
II:5	56	F	*LDLR* +/−	230	-	ECO
II:6	54	F	*LDLR* +/−	227	CAD	-
II:7	50 ^†^	M	*LDLR* +/−	248	-	ECO
II:8	48	M	*LDLR* +/−	201	CAD	-
**III:1**	**32 ^†^**	**M**	***LDLR* +/−*; MYH7* +/−**	**248**	**CAD, LVH**	**-**
III:3	28	M	*LDLR* +/−	278	CAD	-
III:4	27	M	*LDLR* +/−	297	CAD	-
III:5	27	F	*LDLR* +/−	289	CAD	-
III:8	28	M	*LDLR* +/−	211	-	ECO

sLDL = soluble low-density lipoproteins (as from at the time of genotyping); * normal range = <130 mg/dL; CAD = coronary artery disease; F = female; M = male; *LDLR* +/− = heterozygous *LDLR* p.Gly343Cys mutation; *MYH7* +/− = heterozygous *MYH7* p.Arg719Trp mutation; ECO = external carotid occlusion; LVH = left ventricle hypertrophy; ^†^ age of death; mean value over last 5 years; N.A. = not available.

## Data Availability

Data and material are available on reasonable request.
